# Thyroid Hormones, Autoantibodies, Ultrasonography, and Clinical Parameters for Predicting Thyroid Cancer

**DOI:** 10.1155/2016/8215834

**Published:** 2016-05-19

**Authors:** Lin-zheng He, Tian-shu Zeng, Lin Pu, Shi-xiu Pan, Wen-fang Xia, Lu-lu Chen

**Affiliations:** ^1^Department of Endocrinology, Union Hospital, Tongji Medical College, Huazhong University of Science and Technology, Wuhan 430022, China; ^2^Department of Endocrinology, Chengdu First People's Hospital, Chengdu 610041, China

## Abstract

Our objective was to evaluate thyroid nodule malignancy prediction using thyroid function tests, autoantibodies, ultrasonographic imaging, and clinical data. We conducted a retrospective cohort study in 1400 patients with nodular thyroid disease (NTD). The thyroid stimulating hormone (TSH) concentration was significantly higher in patients with differentiated thyroid cancer (DTC) versus benign thyroid nodular disease (BTND) (*p* = 0.004). The receiver operating characteristic curve of TSH showed an AUC of 0.58 (95% CI 0.53–0.62, *p* = 0.001), sensitivity of 74%, and specificity of 57% at a cut-off of 1.59 mIU/L. There was an incremental increase in TSH concentration along with the increasing tumor size (*p* < 0.001). Thyroglobulin antibody (TgAb) concentration was associated with an increased risk of malignancy (*p* = 0.029), but this association was lost when the effect of TSH was taken into account (*p* = 0.11). Thyroid ultrasonographic characteristics, including fewer than three nodules, hypoechoic appearance, solid component, poorly defined margin, intranodular or peripheral-intranodular flow, and punctate calcification, can be used to predict the risk of thyroid cancer. In conclusion, our study suggests that preoperative serum TSH concentration, age, and ultrasonographic features can be used to predict the risk of malignancy in patients with NTD.

## 1. Introduction

The appearance of a thyroid nodule is a frequent occurrence. In the general population, thyroid nodules are found in 4% to 7% of adults through palpation and in 19% to 67% through ultrasonography (US). They are most frequently observed in women and in the elderly, and their prevalence is expected to continue to increase [1, 2]. A large-scale thyroid disease epidemiological investigation in China, the most populous country in the world, has shown that the incidence of thyroid nodules increased from 10.2% in 2006 to 18.6% in 2010.

Although thyroid cancer accounts for only about 1% of all neoplasms, it is the leading cancer site in the endocrine system, and the incidence rate is increasing faster than that of any other malignancy in both men and women, especially differentiated thyroid microcarcinomas (DTMCs), which are tumors ≤1 cm in size. Although DTMCs exhibit a more benign behavior relative to thyroid cancers of larger size (TCLS), there is a subgroup of DTMCs that can be aggressive, requiring therapeutic management similar to TCLS [3].

As a well-established growth factor for thyroid cells, TSH can stimulate the growth of not only normal but also malignant thyroid tissues [4–6]. Current clinical management guidelines emphasize the important role for TSH suppression in the management of patients with high risk thyroid tumors [7, 8]. Recently a number of studies have attempted to address the question of whether TSH exerts an influence on the development of thyroid cancer. A number of studies have shown that serum TSH concentration is an independent risk predictor for the development of thyroid cancer, the progression of thyroid cancer, or both [9–13]. However, there are still some opposing results [14, 15]. Therefore, additional evidence is needed to clarify this question.

In this study, we retrospectively reviewed the records of all patients with one or more thyroid nodules. Our goal was to evaluate thyroid nodule malignancy prediction using thyroid function tests, autoantibodies, US imaging, and clinical data.

## 2. Subjects and Methods

Between June 2008 and December 2010, 1650 patients underwent thyroid surgery for NTD at Wuhan Union Hospital, Tongji Medical College, Huazhong University of Science and Technology. All patients were Chinese nationals, and most of them came from Hubei province in China, where median children urine iodine concentration was higher than 197.5 *μ*g/L between 2005 and 2011. Among these, 1400 patients (men, 267; women, 1133; mean age 47.72 ± 12.69 years) who were confirmed to have a solitary thyroid nodule or a multiple nodules on an ultrasound scan and who were not known to have thyroid cancer or hyperthyroidism due to Graves' disease were included in the present study.

Patients' age, sex, history of taking levothyroxine or antithyroid drugs, preoperative serum TSH concentration, preoperative TPOAb and TgAb concentration, ultrasonographic features, and pathologic data were recorded.

The measurements of serum thyroid function tests were performed with an automated immunochemiluminescent assay (Elecsys 2010, Roche Diagnostics, Manheim, Germany). The normal range for TSH, free triiodothyronine (FT3), and free thyroxine (FT4) was 0.4–4.0 mIU/L (sensitivity 0.01 mIU/L), 12–22 pmol/L (sensitivity 0.01 pmol/L), and 0.27–4.20 pmol/L (sensitivity 0.01 pmol/L), respectively. The measurements of TPOAb and TgAb were also performed with an automated immunochemiluminescent assay (Elecsys 2010, Roche Diagnostics, Manheim, Germany). The normal ranges for TPOAb and TgAb were 0–34 IU/mL and 0–115 IU/mL, and the analytical sensitivities for TPOAb and TgAb were 5 IU/mL and 10 IU/mL, respectively. A titer of greater than the upper limit was defined as positive. Thyroid US was always performed by one of three operators, each with special expertise in thyroid sonography, using HV 900 color HI VISION 900 US system machines (Hitachi Medical, Tokyo, Japan) and a 6–13 MHz linear array transducer.

The final diagnosis of thyroid nodules was dependent on the postoperative histology. Statistical analysis was performed to determinate whether there were differences in the recorded characteristics between patients diagnosed with benign lesions compared with those diagnosed with thyroid cancer.

Differences in the frequencies of single variables were tested with the chi-square test or independent-samples* t*-test. Binary logistic regression analysis was used to identify the independent factors associated with thyroid malignancy. Values were either reported as the mean ± SD or odds ratio (OR) and 95% confidence intervals (CI). In all instances, *p* < 0.05 was considered significant. All data were analyzed using SPSS software for Windows (version 17.0).

## 3. Results

### 3.1. Patients and Tumor Characteristics

The final pathology data showed no evidence of malignancy in 1105 patients (78.9%), whereas malignant lesions were present in 295 patients (21.1%), including 178 papillary thyroid carcinomas, 104 papillary thyroid microcarcinomas, 2 follicular thyroid carcinomas, 4 lymphomas, 2 anaplastic carcinomas, 4 medullary carcinomas, and 4 metastatic carcinomas.

A disproportionate number of women relative to men (1133 : 267) underwent thyroid surgery. Men were more likely to suffer from thyroid cancer than women; 64 of the 267 male patients (23.97%) had malignancy on final pathology* versus* 231 of the 1133 female patients (20.39%), but it was not statistically significant (*p* = 0.197). Patients with malignancy were significantly younger than those without malignancy; the mean age at the time of surgery of the patients with malignancy was 44.33 ± 13.54 years and the mean age of the patients without malignancy was 48.71 ± 12.34 years (*p* < 0.001) (Table 1).

**Figure 1:**
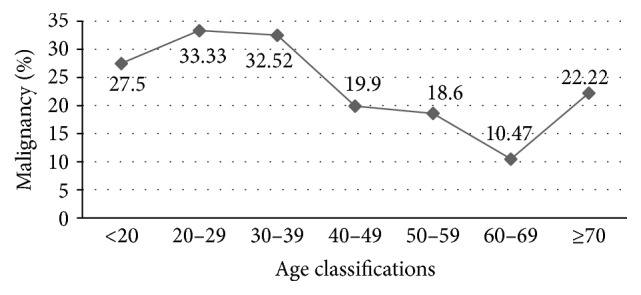
Prevalence of malignancy relative to patient age. Significant increases in the prevalence of malignancy were detected in patients who were younger than 40 years of age (*p* < 0.001, compared with the 40–49-year group) and in those older than 70 years (*p* = 0.036, compared with the 60–69-year group).

Significant increases in the prevalence of malignancy were detected in patients who were younger than 40 years of age (*p* < 0.001, compared with the 40–49-year group) and in those older than 70 years (*p* = 0.036, compared with the 60–69-year group) (Figure 1).

### 3.2. TSH with the Prevalence of DTC

Patients without an available serum TSH concentration within a week before surgery (294 patients), with a final malignancy other than DTC (medullary thyroid cancer, anaplastic thyroid cancer, lymphoma, and metastatic carcinoma, 11 patients), or with a history of taking levothyroxine or antithyroid drugs (129 patients) were not included in the next statistical analysis for TSH. Finally, the remaining 985 patients were eligible for inclusion in the study.

To decrease the likelihood of patients with markedly elevated TSH skewing the data, we excluded all patients with TSH out of the normal range when comparing the mean TSH concentration between different groups. Among the remaining 794 patients, the preoperative mean TSH concentration was significantly higher in patients with DTC* versus* BTND (2.10 ± 0.07 mIU/L* versus *1.86 ± 0.04 mIU/L, *p* = 0.004). If the patients treated with levothyroxine were not excluded, the difference was still statistically significant (*p* = 0.006). In addition, levothyroxine-treated patients with respect to untreated patients showed a marked, but not statistically significant, reduction in the prevalence of DTC (16.98%* versus* 20.48%, *p* = 0.536).

The DTC group was subdivided into DTMCs and TCLS based on the final histological diagnosis. Comparing among the three groups (DTMCs, TCLS, and BTND), the result showed that the patients with BTND had the lowest TSH concentrations, those with DTMCs had intermediate concentrations, and those with TCLS had the highest TSH concentrations (*p* = 0.001). All of the above analyses were repeated after all 149 patients with positive TPOAb or TgAb were removed, and the result did not change (Table 2).

**Figure 2:**
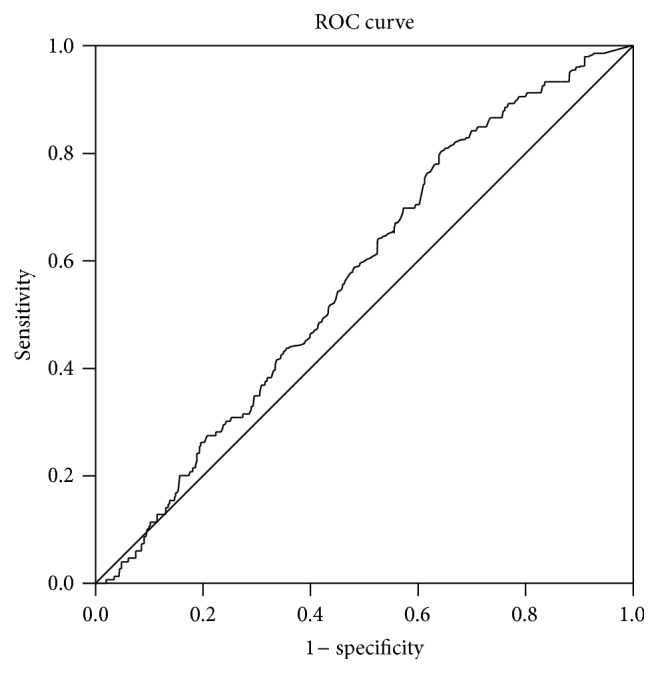
ROC curve for cancer prediction in a model for preoperative TSH testing.

ROC curve analysis was performed to determine the optimal TSH concentration for thyroid cancer prediction. It showed an area under the curve (AUC) of 0.58 (95% CI 0.53–0.62, *p* = 0.001), sensitivity of 74%, and specificity of 57% at a cut-off of 1.59 mIU/L (Figure 2).

**Figure 3:**
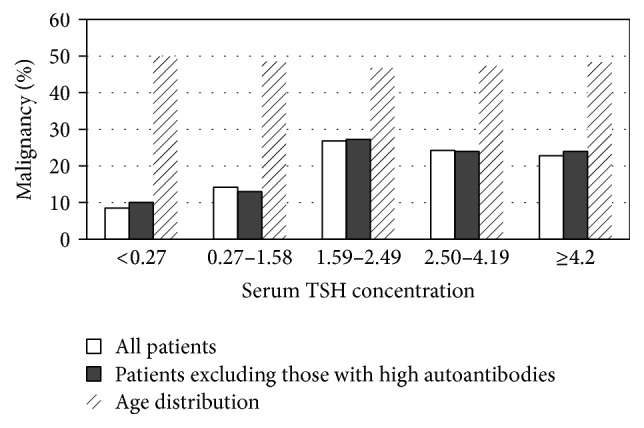
The distribution of patients based on TSH range and historical diagnosis.

Furthermore, the TSH concentration was evaluated as a categorical variable within the following 5 ranges: <0.27 mIU/L (subclinical hyperthyroidism); 0.27–1.58 mIU/L; 1.59 (the cut-off value determined with the ROC curve)–2.50 mIU/L (as more than 95% of normal individuals have TSH levels below 2.5 mIU/L); 2.5–4.19 mIU/L; and ≥4.2 mIU/L (subclinical hypothyroidism). The prevalence of DTC, according to the TSH concentration, indicated a clear TSH-related increase (*p* < 0.001 and *p* = 0.014, resp.). When the patients with positive autoantibodies were removed, the same pattern of escalating cancer incidence with increasing TSH persisted. In addition, among the divided TSH ranges, no significant difference was found between age groups (*p* = 0.339) (Figure 3).

When simultaneously analyzing sex, age, serum TSH concentration, TgAb, and TPOAb with binary logistic regression analysis, the results showed that the risk of DTC was 2.13-fold higher if the TSH level was 1.59 mIU/L or greater relative to TSH levels less than 1.59 mIU/L (Table 3). A simultaneous likelihood ratio test of the effect of all these factors gives *χ* ^2^ = 36.69 (*p* < 0.001), indicating the combination of these factors for the prediction of malignancy to be very valuable.

FT3 and FT4 were also compared between patients with DTC and patients with BTND, but neither of them showed a significant difference, with or without the inclusion of the values out of the normal range (FT3, *p* = 0.77; FT4, *p* = 0.91).

### 3.3. TSH with the Progression of DTC

**Figure 4:**
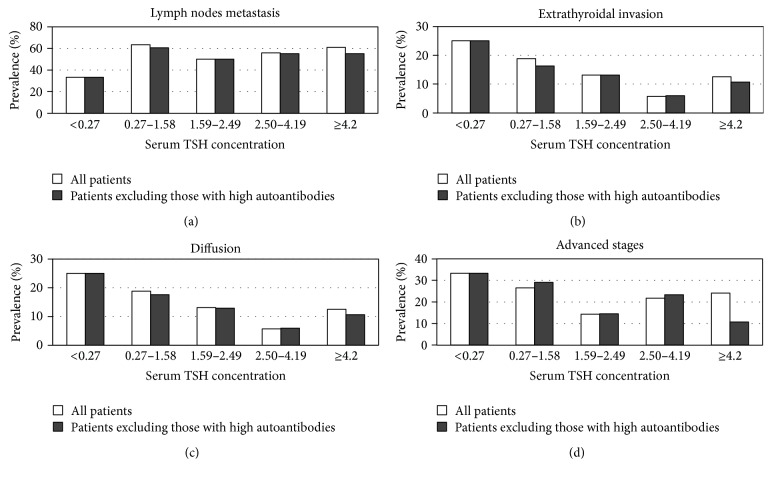
Association of serum TSH concentration and clinicopathological characteristics in differentiated thyroid cancer.

Figure 4 shows the association of serum TSH concentration and pathological characteristics in DTC. The prevalence of lymph node metastasis, extrathyroidal invasion, diffusion (spread in thyroid gland), and advanced stages (stages III and IV, according to the TNM classification) were not related to TSH concentrations (*p* > 0.05). When the patients with positive TPOAb or TgAb were removed from the analysis, the results did not change.

### 3.4. Antibodies and Thyroid Carcinoma

After excluding patients with a history of exposure to levothyroxine or antithyroid drugs, 958 patients had available serum TPOAb results, and 937 had TgAb results.

The prevalence of malignancy was significantly higher in the TgAb-positive group than in the negative group (*p* = 0.029, OR = 1.53, and 95% CI 1.04–2.24). There was a trend toward positive TPOAb and the prevalence of thyroid cancer, but it was not significant (*p* = 0.187), as shown in Table 4. The binary logistic regression analysis (Table 3) indicated that the relationship between elevated TgAb and thyroid cancer did not persist after accounting for other variables (*p* = 0.11, OR = 1.53, and 95% CI 0.91–2.56); the stepwise regression analysis showed that the association was lost when the effect of TSH was taken into account. In addition, TSH values were higher in patients with positive TgAb than in patients without (6.32 ± 1.03 mmol/L* versus *2.43 ± 0.15 mmol/L, *p* < 0.001).

Both positive TPOAb and TgAb were closely correlated with pathologic HT (*p* < 0.001 and *p* < 0.001, resp.). Pathologic HT was detected in 10.92% (31/284) of patients with DTC and 11.0% (121/1105) of patients with BTND, which were not significantly different (*p* = 0.99).

### 3.5. Ultrasonography of Thyroid Carcinoma

Of the 1400 patients who underwent surgery for thyroid nodules between June 2008 and December 2010, 745 were excluded because images could not be retrieved for review. The remaining 655 patients comprised the study set for the evaluation of ultrasonographic features.

Of these 655 patients, 519 (79.2%) patients had benign lesions and 136 (20.8%) had malignant lesions. The prevalence of thyroid cancer did not differ among patients with a solitary thyroid nodule (51 of 236 patients, 21.6%), two nodules (24 of 130 patients, 18.5%), and multiple nodules (61 of 289 patients, 21.1%) (*p* = 0.763).

A total of 374 patients had unilateral thyroid nodules, and the remaining 281 patients had bilateral thyroid nodules. The prevalence of thyroid cancer did not differ between the two groups (81 of 374 patients* versus* 55 of 281 patients, *p* = 0.515).

It was reported that in patients with thyroid cancer in a gland with more than one nodule, 87% had cancer in the largest thyroid nodule [16], so we only recorded the largest nodule in each lobe to analyze the ultrasonographic features. There were 954 nodules in all: 447 nodules from left lobes, 474 from right lobes, and 33 from the isthmus, respectively. There was no significant difference in the thyroid cancer lobe distribution (left lobe, 78 of 447 patients; right lobe, 74 of 474 patients; and isthmus, 8 of 33 patients; *p* = 0.382). The prevalence of thyroid cancer in each lobe did not differ between patients with a solitary nodule and patients with two or more nodules (*p* = 0.538), while those with multiple (≥3) nodules had a lower likelihood of malignancy than those with less than three nodules (*p* = 0.008). Besides the number of nodules, the ultrasonographic characteristics that had a statistically significant association with thyroid cancer included nodular composition (solid component), echogenicity (hypoechoic), poorly defined margin, presence and type of blood flow (intranodular flow and peripheral-intranodular flow), and punctate calcification (Table 5).

### 3.6. Ultrasonography of Thyroid Cancer of the Cervical Lymph Nodes

Using US, the lymph nodes of the neck were evaluated in 512 of 1400 patients. Among these, 440 patients were confirmed to have cervical lymphadenopathy, while the remaining 72 patients did not. The presence of cervical lymphadenopathy was related to a slightly higher prevalence of thyroid cancer (104 of 336 patients, 23.6%* versus* 12 of 72 patients, 16.7%), but the trend was not statistically significant (*p* = 0.19).

There was usually more than one cervical lymph node in each patient, and we only recorded the ultrasonographic characteristics of the lymph node that had the highest likelihood of metastasis in each patient to proceed to the next analysis. The study of ultrasonographic characteristics of cervical lymph nodes and histological diagnoses showed in Table 6.

## 4. Discussion

### 4.1. The Association between Serum TSH and Free Thyroid Hormone Concentrations in BTND and DTC Patients

Since Boelaert et al. [9] reported that TSH could be a risk factor for thyroid cancer in 2006, there have been many subsequent reports supporting their association [9–12, 17–20]. However, some studies made the final diagnosis depending on the FNAB results, which may be subject to ascertainment bias, as some patients may have been misdiagnosed [9, 10, 12, 17, 18, 21]. In some studies, different thyroid malignancies were grouped together, including medullary, anaplastic cancers and thyroid lymphomas, which have never been reported to be TSH dependent [9, 10], and some studies of TSH values have been based on small patient populations [10, 19, 20]. This study largely overcame these limitations by using a large series of patients who underwent thyroid surgery with NTD, successfully further supporting the hypothesis that TSH is a risk predictor for DTC in Chinese patients. In patients who were euthyroid based on TSH levels alone, the mean serum TSH concentrations were significantly higher in patients with DTC compared to those with BTND, regardless of a history of taking levothyroxine. If DTC was subdivided into DTMCs and TCLS, there was an incremental increase in TSH concentration in parallel with the tumor size, which implied that TSH could be not only a predictor of DTC, but also a parameter to determine the size of DTCs. This finding was similar to that in the study by Zafon et al., who found that the increase in TSH levels between the three groups (DTMCs, TCLS, and BTND) of patients was evident, but not statistically significant [22]. If the patients with positive autoantibodies were excluded, the result did not change, implying that the influence of TSH levels on tumorigenesis was not mediated through autoimmunity, corresponding with Fiore et al. [12]. The prevalence of DTC according to patients' TSH concentrations indicated a clear TSH-related increase when we evaluated the TSH concentration as a categorical variable within five ranges, and the incidence of DTC increased significantly when the TSH concentration was higher than 1.59 mIU/L. Our statistics also showed that there was no significant difference in the age distribution among the divided five ranges of TSH concentrations, making it clear that the significant relationship was not based on age. Levothyroxine-treated patients with respect to untreated patients showed a markedly lower prevalence of DTC, although the trend did not reach statistical significance. TSH showed a sensitivity of 74% and specificity of 57% at a cut-off of 1.59 mIU/L determined with ROC curve analysis, which showed an AUC of 0.58 (95% CI 0.53–0.62, *p* = 0.001). When simultaneously analyzing sex, age, TgAb, TPOAb, and serum TSH concentration by binary logistic regression analysis, a 2.13-fold risk of DTC was shown when the TSH concentration was 1.59 mIU/L or greater, relative to TSH concentrations less than 1.59 mIU/L.

Our study failed to show a significant effect of serum TSH concentration on the prognosis of DTC patients. In patients with DTC, the prevalence of lymph node metastases, extrathyroidal invasion, diffusion, and advanced stages (stages III and IV) were not related to TSH concentrations. Some previous research has shown similar results to ours [23]. However, other researchers have shown that higher serum TSH levels were associated with prognostic markers of DTC, including cancer stage, tumor size, lymph node status, extrathyroidal extension, and distant metastases [11, 12, 24]. Clearly, further studies are required as there is still some debate.

In our study, both FT3 and FT4 were not associated with DTC; this result was consistent with some previous studies [12, 14]. Although a prior study has shown that TT3 was associated with DTC [19], the author did not use a separate test for FT3. As the test of TT3 not only reflects the level of FT3 but is also influenced by the level of thyroid binding globulin, this result cannot further demonstrate the association between triiodothyronine and the incidence of thyroid cancer.

### 4.2. Thyroid Antibodies and Thyroid Cancer

In our study, a significantly higher prevalence of cancer was found in patients with serum positive TgAb compared to those with negative TgAb, but not TPOAb, a more specific serum marker of full-blown HT [25]. Unlike some other researches that also showed that TgAb was a predictor for thyroid cancer [23, 26], in our study, the association between TgAb and thyroid cancer no longer existed when the effect of TSH was taken into account. Furthermore, we also showed that the TSH concentration was significantly higher in patients with positive TgAb than in patients with negative TgAb. A previous study showed that the presence of thyroid autoantibodies was associated with a significant increase in TSH, but it did not mention TgAb separately [12]. Based on the results above, TSH may explain the association between positive TgAb and thyroid malignancy; further studies are necessary to clarify this point. The association between TPOAb and thyroid cancer was not found in our study, consistent with some prior studies [9, 27]. It is noteworthy that a large study of palpable thyroid nodules found that TPOAb was associated with an increased risk of malignancy, but this association was lost when the effect of TSH was taken into account [9].

Many studies have discovered a strong association between HT (assessed by the presence of lymphocytic thyroiditis and/or thyroid autoantibodies) and thyroid cancer [28, 29]. It has been reported that there was more lymphocytic thyroiditis in malignant nodules than in benign ones [30]. A recent study also showed that the chronic inflammation that is associated with lymphocytic thyroiditis has the potential to activate cytokines and growth factors and ultimately promote tumorigenesis [31]. Furthermore, HT and PTC share genetic and biomolecular characteristics such as* RET/PTC* rearrangements [32] and the expression of P63 [33] and Akt proteins [34] that are thought to be involved in neoplastic transformation. In addition, the chronic TSH stimulation secondary to HT could be another factor that might promote tumorigenesis. Even so, some other studies opposed the association of HT with thyroid cancer. Particularly, two large prospective studies with a follow-up over 10 years failed to reveal a higher incidence of thyroid cancer in goiters with HT compared with goiters without HT [35, 36]. Our study also showed that HT was not predictive of malignancy through the following two results: (1) TPOAb, a more specific serum marker of full-blown HT, did not show a significant association with thyroid cancer; (2) there was no significant difference in the frequency of pathologic HT in DTC and BTND specimens. Therefore, this result further suggests that the expression of a coexistent HT cannot account for the significantly increased prevalence of cancer in patients with positive TgAb.

### 4.3. US of Thyroid Cancer

As the most common imaging examination for thyroid nodules, US has the advantage of being widely available, well tolerated, affordable, and low-risk. Even though ultrasound alone cannot reliably distinguish malignant and benign lesions, some features have been consistently associated with malignancy according to some researches [37–41]. Our study is the first to investigate the ultrasonographic features of lobe units. There was no significant difference in the prevalence of thyroid malignancy among left lobe, right lobe, and isthmus. By recording the largest nodule in each lobe, our results showed that nodule size was not a predictor of thyroid malignancy. It was reported that a large tumor size indicated an increased risk of malignancy [42–44], while some other researches showed the opposite effect [45, 46]. In addition, a prospective study has shown that increasing nodule size is not predictive of thyroid malignancy [47].

Our results showed that multiple (≥3) nodules had a lower likelihood of malignancy than in cases with less than three nodules, but we failed to show that a single nodule carries an increased risk of thyroid cancer. Previous studies reporting single nodules identified with US [27, 48] showed similar results to ours, while other studies reporting single nodules identified with a physical examination [9] showed results contrary to ours; these differences may be due to the increasing sensitivity of thyroid US, as it was reported that approximately 23% of palpable solitary nodules are actually dominant nodules within a multinodular goiter [49].

Our results also showed that the presence of punctate calcification, solid composition (the more solid a nodule was, the more likely it was to be malignant), hypoechoic regions, and poorly defined margins and the presence and type of blood flow (intranodular flow and peripheral-intranodular flow) were all significant predictors for thyroid cancer, consistent with prior studies [37–41]. The presence of a halo around the nodule was unrelated to the risk of malignancy, consistent with some previous reports [16, 50], but inconsistent with others [51, 52].

### 4.4. US of Cervical Lymph Nodes with Thyroid Cancer

Cervical lymph nodes are involved in a number of disease conditions. For NTD patients, the most common causes of cervical lymphadenopathy are metastasis and reactive lymph nodes. US is increasingly being recognized as a noninvasive tool for the evaluation of cervical lymph nodes. It is known that, in 20%–50% of patients with DTC, the cervical lymph nodes can be involved [53, 54]. Our results showed that the presence of cervical lymphadenopathy in NTD patients was not significantly suggestive of thyroid malignancy (*p* = 0.19), but some ultrasonographic characteristics of lymph nodes were of great significance. A L/S ratio <2, which indicated a more spherical shape, was not only strongly suggestive of malignancy in NTD patients, but it was also suggestive of metastasis in DTC patients, in agreement with prior studies [55, 56]. In addition, the longest diameter ≥15 mm, the shortest diameter ≥5 mm, and the presence of fusion, vascularity, and calcification all indicated the presence of malignancy in NTD patients, but they were not able to differentiate cases with and without metastatic lymph nodes. However, blurred margins were not a distinguishing feature of the lymph nodes in patients with a thyroid malignancy, contrary to what was observed in prior studies [56].

### 4.5. Age and Sex in Thyroid Cancer

Multiple population-based studies have shown age to be an independent risk factor for thyroid cancer [11, 21]; this was confirmed in our study, which revealed that the patients with malignancies were significantly younger compared to those without malignancies. Furthermore, a statistically significant higher rate of malignancy in patients who were under 40 and over 70 years of age was observed in our study. In Western countries, age under 20 and over 70 years was considered a risk factor for thyroid cancer; patients over the age of 20 had a relatively low risk of thyroid cancer compared to patients under the age of 20 years [9]. On the contrary, we found that patients who were 20–40 years of age had a higher risk of thyroid cancer compared to patients younger than 20 years of age. More evidence is needed to clarify whether this discrepancy is caused by population-based differences.

Whether the male sex is a risk factor for thyroid cancer is still controversial. Although a number of research results suggested that men have a higher incidence of thyroid cancer than women do [9–11, 21], some other researches have found that sex did not have a direct impact on the incidence of thyroid cancer [57, 58]; Alexander et al. conducted a follow-up examination 1 month to 5 years after diagnosis for 1009 patients who were treated for benign thyroid nodules, and their result showed that sex did not predict thyroid nodule growth [59]. Our results showed that there was no significant difference in the prevalence of thyroid cancer between men and women.

## Figures and Tables

**Table 1:** Sex and age of thyroid cancer patients.

	Malignancy (%)	No malignancy (%)	*p* value
*N*	295 (21.07%)	1105 (78.93%)	
Sex			0.197
Men	64 (23.97%)	203 (76.03%)	
Women	231 (20.39%)	902 (79.61%)	
Mean age	44.33 ± 13.54 years	48.71 ± 12.34 years	<0.001

**Table 2:** Mean preoperative TSH.

	Number of patients	Mean TSH (mIU/L)	*p* value
All euthyroid patients^a^			
DTC	164	2.10 ± 0.07^b^	0.004^c^
TCLS	111	2.18 ± 0.09	0.001^d^
DTMCs	53	1.94 ± 0.12	
BTND	630	1.86 ± 0.04	
Excluding patients with positive autoantibodies			
DTC	126	2.14 ± 0.08	<0.001^c^
TCLS^b^	85	2.23 ± 0.10	<0.001^d^
DTMCs^c^	41	1.96 ± 0.14	
BTND	519	1.81 ± 0.04	
Excluding patients with HT			
DTC	151	2.11 ± 0.07	0.001^c^
TCLS^b^	104	2.19 ± 0.09	<0.001^d^
DTMCs^c^	47	1.93 ± 0.13	
BTND	586	1.80 ± 0.04	

DTMCs: differentiated thyroid microcarcinomas; TCLS: thyroid cancers of larger size; HT: Hashimoto thyroiditis; BTND: benign thyroid nodular disease; and TSH: thyroid-stimulating hormone. ^a^Euthyroid based on TSH alone; ^b^described as mean ± SE; ^c^comparing group with DTC to group with BTND; ^d^comparing among groups with TCLS, DTMCs, and benign diseases.

**Table 3:** Independent risk factors for the diagnosis of thyroid cancer defined by multivariable logistic regression analysis.

Characteristics	Adjusted odds ratio	95% confidence interval	*p* value
Male sex	1.14	0.75–1.74	0.531
Age	0.98	0.96–0.99	<0.001
TSH ≥ 1.59 mIU/L	2.13	1.48–3.07	<0.001
TPOAb positivity	0.92	0.54–1.57	0.76
TgAb positivity	1.53	0.91–2.56	0.11

TSH: thyroid-stimulating hormone; TPOAb: thyroid peroxidase antibody; and TgAb: thyroglobulin antibody.

**Table 4:** Prevalence of thyroid cancer according to antibodies.

Characteristics	Number of benign lesions (%)	Number of malignant lesions (%)	OR (95% CI)	*p* value
TPOAb			1.30 (0.88–1.92)	0.187
Positivity	134 (76.1%)	42 (23.9%)		
Negativity	630 (80.6%)	152 (19.4%)		
TgAb			1.53 (1.04–2.24)	0.029
Positivity	130 (73.9%)	46 (26.1%)		
Negativity	618 (79.8%)	143 (20.2%)		

OR: odds ratio; CI: confidence interval; TPOAb: thyroid peroxidase antibody; and TgAb: thyroglobulin antibody.

**Table 5:** Ultrasonographic characteristics of thyroid cancer.

Characteristics	Number of benign nodules	Number of malignant nodules	% malignant	*p* value
Number of nodules				0.024
1	404	93	18.7	0.538^a^
2	89	24	21.2	0.008^b^
≥3	301	43	12.5	
Size, mm (mean ± SE)	21.861 ± 0.485	23.295 ± 1.198		0.235
Composition				<0.001
Completely solid	387	122	24.0	
Predominantly solid	200	27	11.9	
Predominantly cystic	131	6	4.4	
Completely cystic	76	5	6.2	
Echogenicity				<0.001
Hypoechoic	273	100	26.8	
Hyperechoic	111	21	15.9	
Isoechoic	65	5	7.1	
Mixed echoic	233	45	16.2	
Anechoic	94	7	6.9	
Margin				<0.001
Poorly defined	136	68	33.3	
Well defined	640	110	14.7	
Blood flow				<0.001
Absent	189	23	10.8	
Peripheral	113	14	11.0	
Intranodular	311	99	24.1	
Peripheral-intranodular	164	41	20.0	
Calcification				<0.001
None	517	80	13.4	
Punctate	200	82	29.1	
Coarse	59	16	21.3	
Halo				0.109
None	761	171	18.3	
Present	15	7	31.8	

^a^Comparison between the group with a single nodule to the group with 2 or more nodules.

^b^Comparison between the group with <3 nodules to the group with ≥3 nodules.

**Table 6:** Ultrasonographic characteristics of cervical lymph nodes and histological diagnoses.

Ultrasonographic characteristics	Histological diagnosis of thyroid nodules				Lymph nodes metastasis			
	Benign	Malignant	OR (95% CI)	*p*	Present	Absent	OR (95% CI)	*p*
Longest diameter			2.55 (1.47–4.42)	0.001			1.86 (0.54–6.44)	NS
≥15 mm	191	77			40	27		
<15 mm	120	19			11	4		
Shortest diameter			2.08 (1.28–3.37)	0.003			0.84 (0.31–2.27)	NS
≥5 mm	160	66			38	22		
<5 mm	151	30			13	9		
L/S ratio^a^			4.11 (2.14–7.87)	<0.001			3.38 (1.02–11.21)	0.04
>2	290	74			17	4		
<2	21	22			34	27		
Margins			3.34 (0.47–24.04)	NS			1.66 (0.1–27.41)	NS
Blurred	2	2			1	1		
Defined	331	99			53	32		
Fusion			24.80 (3.01–204.08)	<0.001			1.70 (1.42–2.05)	0.031
Present	1	7			7	0		
Absent	333	94			47	33		
Vascularity			2.03 (1.26–3.26)	0.003			0.59 (0.24–1.48)	NS
Present	176	70			39	20		
Absent	158	31			15	13		
Calcification			48.39 (6.25–374.84)	<0.001			0.28 (0.06–1.39)	NS
Present	0	12			10	2		
Absent	334	89			44	31		

^a^L/S ratio: large axis to small axis ratio.

## Additional Points

We acknowledge the following limitations of this study: (1) retrospective studies of pathology may be subject to ascertainment bias; (2) in our study, only the patients who underwent surgery for NTD were included, creating a selection bias, as surgery was only performed for the NTD patients whose nodules were considered to have the possibility of malignancy; and (3) we only recorded the largest thyroid nodules in each thyroid lobe and the lymph node that had the greatest likelihood of metastasis based on US, so we have inevitably missed some important information.
